# Association of frailty with outcomes of resection for colonic volvulus: A national analysis

**DOI:** 10.1371/journal.pone.0276917

**Published:** 2022-11-08

**Authors:** Shayan Ebrahimian, Cory Lee, Zachary Tran, Sara Sakowitz, Syed Shahyan Bakhtiyar, Arjun Verma, Areti Tillou, Peyman Benharash, Hanjoo Lee

**Affiliations:** 1 Cardiovascular Outcomes Research Laboratories, Division of Cardiac Surgery, David Geffen School of Medicine at UCLA, Los Angeles, CA, United States of America; 2 Department of Surgery, Loma Linda University Medical Center, Loma Linda, CA, United States of America; 3 Department of Surgery, University of Colorado, Aurora, CO, United States of America; 4 Department of Surgery, David Geffen School of Medicine at UCLA, Los Angeles, CA, United States of America; 5 Department of Surgery, Harbor-UCLA Medical Center, Torrance, CA, United States of America; Charité Universitätsmedizin Berlin CVK: Charite Universitatsmedizin Berlin - Campus Virchow-Klinikum, GERMANY

## Abstract

**Background:**

With limited national studies available, we characterized the association of frailty with outcomes of surgical resection for colonic volvulus.

**Methods:**

Adults with sigmoid or cecal volvulus undergoing non-elective colectomy were identified in the 2010–2019 Nationwide Readmissions Database. Frailty was identified using the Johns Hopkins indicator which utilizes administrative codes. Multivariable models were developed to examine the association of frailty with in-hospital mortality, perioperative complications, stoma use, length of stay, hospitalization costs, non-home discharge, and 30-day non-elective readmissions.

**Results:**

An estimated 66,767 patients underwent resection for colonic volvulus (Sigmoid: 39.6%; Cecal: 60.4%). Using the Johns Hopkins indicator, 30.3% of patients with sigmoid volvulus and 15.9% of those with cecal volvulus were considered frail. After adjustment, frail patients had higher risk of mortality compared to non-frail in both sigmoid (10.6% [95% CI 9.47–11.7] vs 5.7% [95% CI 5.2–6.2]) and cecal (10.4% [95% CI 9.2–11.6] vs 3.5% [95% CI 3.2–3.8]) volvulus cohorts. Frailty was associated with greater odds of acute venous thromboembolism occurrences (Sigmoid: AOR 1.50 [95% CI 1.18–1.94]; Cecal: AOR 2.0 [95% CI 1.50–2.72]), colostomy formation (Sigmoid: AOR 1.73 [95% CI 1.57–1.91]; Cecal: AOR 1.48 [95% CI 1.10–2.00]), non-home discharge (Sigmoid: AOR 1.97 [95% CI 1.77–2.20]; Cecal: AOR 2.56 [95% CI 2.27–2.89]), and 30-day readmission (Sigmoid: AOR 1.15 [95% CI 1.01–1.30]; Cecal: AOR 1.26 [95% CI 1.10–1.45]). Frailty was associated with incremental increase in length of stay (Sigmoid: +3.4 days [95% CI 2.8–3.9]; Cecal: +3.8 days [95% CI 3.3–4.4]) and costs (Sigmoid: +$7.5k [95% CI 5.9–9.1]; Cecal: +$12.1k [95% CI 10.1–14.1]).

**Conclusion:**

Frailty, measured using a simplified administrative tool, is associated with significantly worse clinical and financial outcomes following non-elective resections for colonic volvulus. Standard assessment of frailty may aid risk-stratification, better inform shared-decision making, and guide healthcare teams in targeted resource allocation in this vulnerable patient population.

## Introduction

With increasing availability of treatment options and emphasis on patient-centered care, a pragmatic discussion of surgical risks and long-term outcomes is needed [1]. This point is particularly relevant in high risk surgeries. Colonic volvulus accounts for 1.9% - 3.4% of bowel obstructions in the United States and is associated with high morbidity and overall mortality [2–4]. However, factors influencing outcomes of surgical intervention in patients with colonic volvulus requiring resection remain understudied at the national level [2–4].

Traditional risk factors aside, a large body of literature has demonstrated frailty to be a strong, independent marker for increased mortality and perioperative complications [5–10]. Frailty is a multifaceted clinical syndrome characterized as a state of physical and cognitive decline, increased vulnerability to stressors and social withdrawal [11, 12]. Several studies have shown frailty to be a superior predictor of surgical risk compared to the historically used patient age and preoperative comorbidities [13, 14]. Although numerous assessment tools have been used to quantify frailty, they are resource intensive and difficult to adopt [15–18]. The Johns Hopkins Adjusted Clinical Groups (ACG) frailty indicator has been introduced to address these limitations. As a coding-based instrument, the ACG-frailty indicator can be readily used in the clinical setting and has been validated in a multitude of surgical specialties [5–10].

The objective of the current study was to characterize the association of frailty, as assessed by the Johns Hopkins frailty index, with clinical outcomes and resource use following non-elective resection for colonic volvulus. We hypothesized that patients classified as Frail would experience increased risk of mortality, perioperative complications, non-home discharge, as well as greater hospitalization costs and longer length of stay (LOS).

## Materials and methods

### Data source and study population

This was a retrospective cohort study using the 2010–2019 Nationwide Readmission Database (NRD). Maintained as part of the Healthcare Cost and Utilization Project (HCUP), the NRD is the largest all-payer, readmissions database and uses specific hospital-based discharge weights to accurately estimate approximately 60% of hospitalizations in the United States [19]. The NRD contains unique patient and hospital identifiers allowing hospital admissions to be tracked within each calendar year.

**Fig 1:**
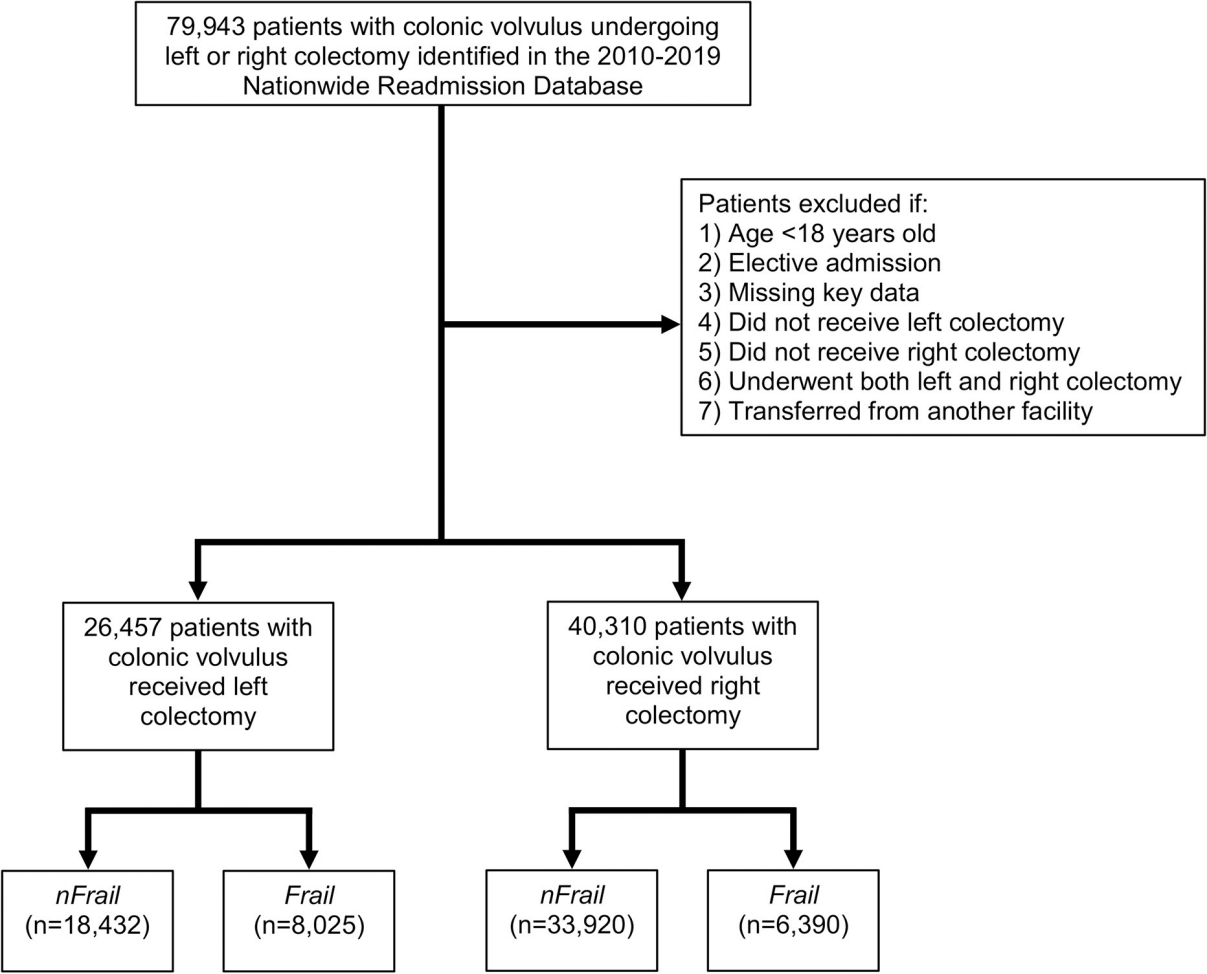
Flow chart of the patient population selection process.

All adults (≥ 18 years) with a diagnosis of colonic volvulus were identified using the *International Classification of Diseases*, *Ninth and Tenth Revisions* (ICD-9/10) diagnosis codes (560.2, K56.2) [2]. Patients undergoing non-elective left or right colectomy were included for further analysis (S1 Table). The ICD-9/10 procedure codes for left and right colectomy were utilized to delineate between sigmoid and cecal volvulus [2]. Records with elective admissions, transfers from another facility, codes for both left and right colectomy, or missing data for age, sex, in-hospital mortality, and hospitalization costs were excluded (2.2% missing key data; Fig 1). Patients were classified as *Frail* using ICD-9/10 diagnosis codes associated with conditions in the Johns Hopkins ACG cluster, which included malnutrition, weight loss, falls, dementia, difficulty walking, impaired vision and incontinence, among others [7, 20]. The remaining patients comprised the non-frail cohort (*nFrail)*.

### Variable definitions and study outcomes

Patient and hospital characteristics were defined according to the HCUP data dictionary and included age, sex, income quartile, and insurance payer as well as hospital teaching status and bed size [19]. The Van Walraven modification of the Elixhauser Comorbidity Index, a validated composite of 30 comorbidities, was utilized to quantify the burden of chronic conditions [21]. Individual comorbidities and complications, such as perioperative hemorrhage, postoperative infections, and acute venous thromboembolism (VTE) were tabulated using previously published ICD-9/10 codes [8, 22–24]. The diagnosis of VTE was ascertained using codes for acute pulmonary embolism and deep venous thrombosis [23]. Hospitalization costs were calculated using HCUP hospital specific cost-to-charge ratios and inflation adjusted to 2019 Personal Health Care Price Index [19]. The ICD-10 procedure code for colonoscopy (0DJD8ZZ) was utilized to identify endoscopic detorsion attempts. Resections within one day of admission were classified as emergent while failure of endoscopic detorsion was defined as an attempted endoscopic decompression on the same day as colectomy. To determine timing to surgical intervention, 2016–2019 years were utilized due to inconsistent reporting of procedure date in prior years.

#### Study objective

The primary outcome of this study was in-hospital mortality during index admission while secondary endpoints included perioperative hemorrhage, postoperative infections, acute VTE, colostomy or ileostomy occurrences, length of stay, index hospitalization costs, non-home discharge, and 30-day non-elective readmission.

#### Statistical analysis

Categorical variables are reported as proportion and compared using the Pearson’s Χ^2^ test. Continuous variables are summarized as medians with interquartile range [IQR] and compared using Mann Whitney U tests. Cuzick’s non-parametric test of trend (nptrend) was utilized to assess the changes in mortality rates and proportion of frail patients over the 10-year period [25]. Multivariable logistic and linear regression models were developed to evaluate the association of frailty with outcomes of interest. Model covariates were determined using elastic net regularization, an automated method utilized for variable selection and to increase out-of-sample generalizability [26]. Regression outputs are reported as adjusted odds ratios (AOR) for logistic and as beta coefficients (β) for linear models with 95% confidence intervals (AOR/ β 95% CI). Statistical significance was defined as α<0.05, and all analyses were performed using Stata software version 16.0 (StataCorp LP, College Station, TX). This study was deemed exempt from full review by the Institutional Review Board (IRB) at the University of California, Los Angeles (IRB# 17–001112). Patient consent was also waived due to the deidentified nature of the NRD.

## Results

### Demographic comparison

An estimated 66,767 patients underwent a sigmoid (39.6%) or cecal (60.4%) resection for colonic volvulus (Fig 1). Using the Johns Hopkins ACG classification, 30.3% of patients with sigmoid volvulus and 15.9% of those with cecal volvulus were considered *Frail*. The distribution of frailty-related diagnoses is shown in Table 1. Malnutrition and dementia were the most common frailty-associated clusters identified among *Frail* patients (Table 1). Patient, operative, and hospital characteristics of sigmoid and cecal volvulus cohorts grouped by frailty are summarized in Table 2. Compared to *nFrail*, patients categorized as *Frail* among both sigmoid and cecal volvulus cohorts were older, had a higher burden of comorbidities as measured by Elixhauser comorbidity index, and more commonly classified in the lowest income quartile (Table 2). While *Frail* patients with cecal volvulus were more commonly male, the distribution of sexes was similar among *Frail* and *nFrail* cohorts with sigmoid volvulus (Table 2). Laparoscopic operative approach was less frequently deployed among *Frail* patients in both sigmoid and cecal volvulus cohorts (Table 2).

**Table 1:** Johns Hopkins Adjusted clinical Groups frailty-defining diagnosis clusters.

Cluster	Examples of ICD-9 and ICD-10 Diagnoses	Sigmoid Volvulus (%)	Cecal Volvulus (%)
Malnutrition	Nutritional marasmus; other severe protein-calorie malnutrition	33.2	51.9
Dementia	Presenile dementia; vascular dementia	38.6	25.5
Blindness	Profound impairment in both eyes; legal blindness as defined in the United States	1.6	1.9
Pressure ulcer	Decubitus ulcer; pressure ulcer, unspecified site	15.2	8.0
Urinary Incontinence	Atony of bladder; continuous leakage	0.6	0.2
Weight loss	Anorexia; feeding difficulties and mismanagement	5.7	8.6
Fecal incontinence	Incontinence of feces	1.8	0.9
Homelessness	Lack of housing; inadequate materials resources, no other household member able to render care	1.1	1.3
Gait	Abnormality of gait; lack of coordination	1.7	1.1
Fall	Fall from chair; history of fall	0.5	0.6

ICD-9. International Classification of Diseases, Ninth Revision

ICD-10. International Classification of Diseases, Tenth Revision

**Table 2:** Comparison of patient, operative, and hospital-level characteristics of sigmoid and cecal volvulus cohorts grouped by frailty.

Parameter	Sigmoid Volvulus		*P*	Cecal Volvulus		*P*
	*nFrail* (n = 18,432)	*Frail* (n = 8,025)		*nFrail* (n = 33,920)	*Frail* (n = 6,390)	
**Patient characteristics**						
Age (years, median, IQR)	70 [57–80]	77 [67–84]	<0.001	64 [53–75]	75 [64–83]	<0.001
Female (%)	33.5	31.6	0.08	76.8	66.2	<0.001
Income quartile (percentile) (%)			0.03			<0.001
76^th^-100^th^	22.7	20.3		27.7	20.8	
51^st^-75^th^	24.7	24.3		27.0	26.1	
26^th^-50^th^	25.2	25.6		24.8	26.5	
0-25^th^	27.4	29.7		20.6	26.4	
Insurance type (%)			<0.001			<0.001
Private	18.1	6.6		36.0	12.7	
Medicare	69.4	83.4		52.5	76.0	
Medicaid	8.1	7.3		6.3	7.4	
Other	4.4	2.7		5.2	3.9	
Elixhauser Comorbidity Index (median, IQR)	3 [2–4]	4 [3–5]	<0.001	2 [1–4]	4 [3–6]	<0.001
Comorbidities (%)						
Congestive heart failure	13.7	18.6	<0.001	7.9	17.2	<0.001
Coronary artery disease	16.6	19.3	<0.002	12.0	17.8	<0.001
Cardiac Arrhythmia	27.7	35.8	<0.001	20.2	33.4	<0.001
Hypertension	55.3	59.6	<0.001	45.4	53.6	<0.001
Tobacco use	5.9	3.8	<0.001	10.6	9.2	0.049
Pulmonary circulatory disorders	2.8	3.3	0.16	2.4	4.4	<0.001
Chronic pulmonary disease	16.5	18.4	0.02	20.1	27.3	<0.001
Peripheral vascular disorder	11.4	13.8	<0.001	11.6	15.9	<0.001
Diabetes	18.7	21.9	<0.001	11.5	15.1	<0.001
Liver Disease	2.9	3.7	0.02	3.3	4.6	0.001
Coagulopathy	7.0	10.1	<0.001	4.4	9.3	<0.001
Anemia	4.3	5.7	0.002	3.4	5.4	<0.001
Peptic ulcer disease	0.6	1.0	0.04	0.5	0.9	0.02
Cancer	3.7	4.6	0.02	4.2	7.8	<0.001
Obesity	7.8	4.9	<0.001	5.3	3.7	0.001
Fluid and electrolyte disorders	55.6	72.5	<0.001	39.4	65.9	<0.001
End stage renal disease	10.6	14.0	<0.001	6.7	11.6	<0.001
**Operative characteristics**						
Colectomy approach (%)			<0.001			<0.001
Open	87.2	91.2		92.7	96.7	
Laparoscopic	12.8	8.8		7.3	3.3	
**Hospital characteristics**						
Hospital bed size			0.4			<0.001
Small	15.2	15.1		17.7	14.8	
Medium	26.3	27.7		27.8	27.1	
Large	58.5	57.3		54.5	58.1	
Location/Teaching Status			0.7			0.6
Non-metropolitan	10.4	10.8		11.2	11.2	
Metropolitan non-teaching	31.1	31.7		32.7	31.6	
Metropolitan teaching	58.4	57.6		56.1	57.2	

Compared to *nFrail*, *Frail* patients in the sigmoid volvulus cohort experienced similar rates of emergency resection, endoscopic detorsion attempts, and failure of endoscopic detorsion (Table 3). The rates of endoscopic detorsion attempts as well as failure of endoscopic detorsion were similar between *Frail* and *nFrail* patients in the cecal volvulus cohort (Table 3). *Frail* patients with cecal volvulus less frequently underwent emergent resection, compared to their non-frail counterparts (Table 3). The median interval from admission to non-emergent resection was longer among *Frail* patients in both sigmoid and cecal volvulus cohorts (Table 3).

**Table 3:** Operative and endoscopic detorsion characteristics among patients with sigmoid and cecal volvulus undergoing resection during 2016–2019 stratified by frailty.

Parameter	Sigmoid Volvulus		*P*	Cecal Volvulus		*P*
	*nFrail* (8,064)	*Frail* (n = 3,774)		*nFrail* (n = 14,751)	*Frail* (n = 2,939)	
Emergent resection (%)	44.8	43.3	0.3	87.6	75.9	<0.001
Interval to non-emergent resection (days, median, IQR)	3 [2–5]	4 [3–7]	<0.001	3 [2–6]	4 [2–7]	0.0015
Endoscopic detorsion (%)	26.8	26.4	0.7	2.9	3.8	0.08
Failure of endoscopic detorsion (%)	10.1	9.8	0.7	1.4	1.4	0.85

Emergent resection was defined as colectomy that occurred within one day of admission.

Failure of endoscopic detorsion was defined as an attempted endoscopic decompression on the same day as resection.

### Outcomes of patients with sigmoid volvulus

**Fig 2:**
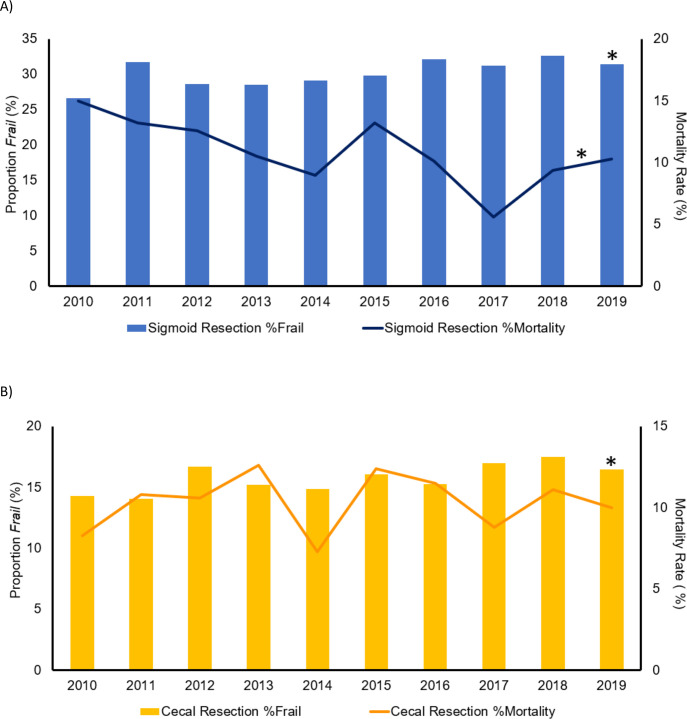
Temporal trends in prevalence of frailty among patients with sigmoid (A) or cecal (B) volvulus undergoing non-elective resection and the associated in-hospital mortality rates in *Frail* cohort from 2010 to 2019. The bar graphs depict the annual proportion of patients undergoing sigmoid (blue bars) or cecal (yellow bars) volvulus resection who were considered *Frail*. The line graphs represent the annual, unadjusted in-hospital mortality rates among *Frail* patients who receive sigmoid (dark blue line) or cecal volvulus resection (orange line). *The non-parametric test of trend (nptrend), *P* <0.05.

While the prevalence of frailty in the sigmoid volvulus cohort increased from 26.6% in 2010 to 31.4% in 2019 (nptrend = 0.007), unadjusted in-hospital mortality rates among *Frail* patients declined from 15% to 10% during the same period (nptrend = 0.008; Fig 2A). Nonetheless, *Frail* patients undergoing left colectomy faced higher unadjusted rates of in-hospital mortality, postoperative infections, and VTE occurrence, compared to *nFrail* (S2 Table). Colostomy formation was more common among *Frail* relative to *nFrail*, and rates of ileostomy and perioperative hemorrhage were similar among the two groups. The *Frail* cohort faced a longer LOS, greater index hospitalization costs, as well as higher rates of non-home discharge and 30-day non-elective readmission compared with *nFrail* (S2 Table).

Following adjustment of intergroup differences (C-statistic: 0.76), frailty was independently associated with greater odds of in-hospital mortality (AOR 1.31, 95% CI 1.09–1.56) with an absolute increment in risk from 5.7% (95% CI 5.2–6.2) to 10.6% (95% CI 9.47–11.7). Frailty also remained associated with greater odds of postoperative infections and VTE occurrence (Table 4). Although frailty did not alter the risk of ileostomy or perioperative hemorrhage, it was associated with increased odds of colostomy. Moreover, frailty was independently associated with incremental increases in LOS and index hospitalization costs. Frailty was also linked to greater odds of non-home discharge and 30-day non-elective readmission (Table 4).

**Table 4:** Multivariable risk association of perioperative outcomes among *Frail* patients (reference: *nFrail*) with sigmoid and cecal volvulus undergoing resections.

Parameter	Sigmoid Volvulus		Cecal Volvulus	
	AOR or β-coefficient	95% CI	AOR or β-coefficient	95% CI
**Clinical Outcomes**				
In-hospital mortality	1.31	1.09–1.56	1.51	1.24–1.85
Perioperative hemorrhage	0.75	0.53–1.07	1.21	0.87–1.69
Postoperative infections	1.30	1.02–1.67	1.32	1.03–1.69
Venous Thromboembolism	1.50	1.18–1.94	2.0	1.50–2.72
Colostomy	1.73	1.57–1.91	1.48	1.10–2.00
Ileostomy	0.80	0.61–1.05	1.94	1.63–2.29
**Resource Utilization**				
Length of stay (days)	3.4	2.8–3.9	3.8	3.3–4.4
Index hospitalization costs ($1,000s)	7.5	5.9–9.1	12.1	10.1–14.1
Non-home discharge	1.97	1.77–2.20	2.56	2.27–2.89
30-day non-elective readmission	1.15	1.01–1.30	1.26	1.10–1.45

Associations reported as adjusted odds ratio (AOR) (mortality, complications, non-home discharge, and readmission) or β-coefficient (costs and length of stay). All models were adjusted for demographics (age, sex, income, primary payer), Elixhauser index, comorbidities (congestive heart failure, coronary artery disease, cardiac arrythmia, hypertension, tobacco use, pulmonary circulatory disorders, chronic pulmonary disease, peripheral vascular disorder, diabetes, liver disease, coagulopathy, anemia, peptic ulcer disease, cancer, obesity, fluid and electrolyte disorders, and renal failure), colectomy approach (open vs. laparoscopic), and hospital characteristics (hospital bed size and location/teaching status).

### Outcomes of patients with cecal volvulus

The prevalence of frailty in the cecal volvulus cohort increased from 14.3% to 16.5% (nptrend <0.001) without changes in the in-hospital mortality rates over the 10-year period (nptrend = 0.77; Fig 2B). Overall, *Frail* patients with cecal volvulus undergoing resection faced greater rates of unadjusted in-hospital mortality, perioperative hemorrhage, postoperative infections, and VTE occurrence compared to *nFrail* patients (S2 Table). Rates of both ileostomy and colostomy were higher among *Frail* patients compared to their *nFrail* counterparts. *Frail* patients also experienced a longer LOS, greater index hospitalization costs, as well as higher rates of non-home discharge and 30-day non-elective readmission compared with the *nFrail* group (S2 Table).

After adjustment (C-statistic = 0.85), frailty remained independently associated with increased odds of in-hospital mortality (AOR 1.51, 95% CI 1.24–1.85) among the cecal volvulus cohort, which translated into approximately two-fold increase in predictive risk of mortality (*Frail*: 10.4%, 95% CI 9.2–11.6; *nFrail*: 3.5, 95% CI 3.2–3.8). Additionally, frailty portended greater odds of postoperative infections and VTE occurrence, as well as both ileostomy and colostomy. However, frailty was no longer associated with perioperative hemorrhage complications. Frailty exhibited a significant association with LOS, hospitalization costs, non-home discharge, and 30-day non-elective readmission (Table 4).

## Discussion

In the present work, we examined the association of frailty, as measured by an administrative tool, with outcomes of surgical treatment for colonic volvulus and made several important observations. Utilizing the John Hopkins ACG frailty indicator, approximately 30% of patients with sigmoid volvulus and 16% of those with cecal volvulus were considered *Frail*. Frailty was associated with a near doubling in the risk of in-hospital mortality among both sigmoid and cecal volvulus cohorts. Moreover, frailty was associated with greater use of resources as *Frail* patients in both sigmoid and cecal volvulus cohorts were at increased odds of stoma formation, non-home discharge, and 30-day non-elective readmission, as well as longer risk-adjusted LOS and greater hospitalization costs. Several of these findings merit further discussion.

A large body of literature has demonstrated frailty to consistently portend inferior postoperative outcomes in both emergent and elective settings, irrespective of the assessment method [5–10]. However, detailed frailty assessment tests such as gait speed and grip strength may be impractical in the emergent settings. Therefore, use of administrative coding algorithms, such as the John Hopkins ACG frailty indicator, may provide a practical alternative to identify frailty in non-elective operations. To date, the utility of the ACG frailty indicator among patients with colonic volvulus remains undefined. This is especially pertinent considering large bowel obstruction due to sigmoid and cecal volvulus generally occurs in the 6^th^ and 7^th^ decades of life and in patients who may suffer from various other comorbidities [2]. Our findings suggest that the ACG frailty indicator is an effective tool to predict mortality, perioperative complications as well as index-hospital and post-discharge expenditure among patients with sigmoid and cecal volvulus undergoing non-elective resection. Thus, the incorporation of administrative coding algorithms into electronic medical records may guide postoperative recovery strategies to optimize outcomes in frail patients with colonic volvulus.

Although frailty may not be considered a preoperative modifiable risk factor in an acute setting, knowledge of its presence identifies patients who may benefit from targeted resource allocation. Our findings suggest that frailty is associated with increased healthcare costs and rates of non-home discharge. Longer hospitalizations and higher costs attributed to frailty may in part, reflect the time required to manage comorbidities and coordinate transitional care prior to discharge. Notably, preoperative cognitive impairment, a commonly diagnosed frailty criterion in our study, is a known risk factor for postoperative delirium [27]. This in turn is associated with increased morbidity, hospitalization costs, and non-home discharge [28–31]. Therefore, close monitoring of cognitive status of these patients in the perioperative period is imperative. While independently associated with inferior perioperative outcomes, the frailty-defining diagnoses, such as malnutrition, dementia, weight loss, and pressure ulcers, may also perpetuate the existence of one another [32, 33]. Thus, frail patients may benefit from a multidisciplinary approach to their perioperative care. The slightly lower rates of emergent resection and delayed median interval from admission to non-emergent resection among *Frail* patients (Table 3) may, in fact, reflect multidisciplinary efforts to optimize patients prior to committing to potentially morbid surgery.

In addition to such multidisciplinary management strategies, early recovery care after surgery (ERAS) program in the perioperative period should be strongly considered. ERAS for colorectal surgery among elderly patients has demonstrated equivalent benefits of reducing postoperative morbidity to other age groups [34]. However, a more personalized ERAS program for frail patients would be sensible. For example, fluid management may be individualized in frail patients with congestive heart failure, acute kidney injury, and those who have received mechanical bowel preparation [1]. Mechanical bowel preparation should only be attempted once the volvulus has been relieved with endoscopic detorsion [1]. It then serves as a dual function of cleansing the bowel content thereby reducing risk of surgical site infection and decompressing the proximal bowel in preparation for an anastomosis to the diminutive distal bowel. Moreover, the benefits and safety of laparoscopic surgery have recently been explored for colorectal surgery among frail patients [35–37]. Similar advantages of laparoscopic surgery, such as decreased surgical site infection, opioid use, time to return of bowel function, and length of hospitalization, can be enjoyed in frail patients as in the general population [35–37]. Although laparoscopic surgery was less frequently used in frail patients compared to non-frail individuals in our study, it should be considered whenever expertise is available.

Social frailty is a relatively novel concept exploring the intimate relationship between a patient’s socioeconomic factors, physical environment, and access to care–so-called social determinants of health (SDoH)—and frailty [38]. The American Society of Colon and Rectal Surgeons recently published a guideline with a recommendation to screen for social frailty in elderly frail patients and provide an appropriate support system to encourage healthy behavior changes in their environment [1]. Understanding social frailty is especially germane in colon surgery as colonic pathologies such as volvulus often involve elderly, frail patients. Inadequate access to healthy nutrition and lack of reliable transportation for follow-up care may increase the risk for poor outcomes after colon surgery [39]. Therefore, coordination of care along with social work early in the hospital course would be prudent [40].

The cornerstone of managing frail patients is understanding the patient’s goal of care within the context of their frailty and overall prognosis [1]. This entails a multidisciplinary care team consisting of geriatrics and palliative services in coordination with the patient and their family members. The discussion should address the patient’s longevity, functional status, and how the proposed management, including surgical options, may affect these factors [1]. It may be wise for the managing team to recognize that frail patients may measure treatment success differently based on their goals. In this regard, management course may inevitably deviate from standard of care [40]. The increased rate of ostomy construction after colectomy in the current study may partly reflect the coordinated decision between the surgeon and the patient to exhaust every option to minimize any potential anastomotic leakage and its associated morbidity. Thus, increased rates of colostomy and ileostomy in our study should not necessarily be viewed as inferior outcomes, and surgeons should carefully consider ostomy construction if it aligns with patients’ goals of care.

The present study has several important limitations. Due to the observational nature of the study design, causal inferences cannot be drawn. Although the NRD is the largest all-payer readmission database, it is subjected to variations in coding practices between physicians and institutions. The exact anatomy of the volvulized segment of intestine could not be delineated as only one ICD-9/10 diagnostic code is available for colonic volvulus. While we utilized ICD-9/10 procedure codes for left and right colectomy to ascertain the presence of sigmoid or cecal volvulus, the extent of resections and the presence of anastomosis could not be determined. Clinical data such as laboratory values, imaging features, and measures of disease severity were unavailable for analysis. Additional clinical markers of frailty such as hypoalbuminemia, sarcopenia, and grip strength could not be captured. Moreover, as outpatient mortality data and postoperative complications after index-hospitalization are unavailable in the NRD, we limited our analysis to post-discharge healthcare utilization, including non-home discharge and readmissions. Therefore, we were unable to comment on the impact of frailty on long-term outcomes such as post discharge mortality and complications associated with creation and closure of ostomy. Despite these limitations, we utilized the largest all-payer national database and adhered to appropriate data practices as recommended by HCUP to report nationally representative outcomes.

## Conclusion

Frailty as measured by a coding-based binary tool was independently associated with inferior outcomes among patients with colonic volvulus undergoing resection. Particularly frailty imparted a near doubling in the predicted risk of mortality as well as a proportionally greater detriment in index-hospital and post-discharge expenditure. Simple frailty assessments preoperatively may inform expectations, identify high-risk patients, and guide healthcare systems in targeted resource allocation to optimize outcomes in this vulnerable patient population.

## Supporting information

S1 Table International Classification of Diseases, Ninth and Tenth Revision (ICD-9/10) procedural codes used to identify left and right colectomy. (DOCX)

S2 Table Unadjusted outcomes for patients with sigmoid and cecal volvulus undergoing resection stratified by frailty. (DOCX)
