# Supplementary material for: Association of frailty with outcomes of resection for colonic volvulus: A national analysis
Source: PLoS One. 2022 Nov 8;17(11):e0276917. doi: 10.1371/journal.pone.0276917 (PMC9642887; doi:10.1371/journal.pone.0276917)
Supplement: S1 Table — (DOCX) [file pone.0276917.s001.docx]

**S1 Table. International Classification of Diseases, Ninth and Tenth Revision (ICD-9/10) procedural codes used to identify left and right colectomy**

| **Operation** | | **Code type** | **Code** |
| --- | --- | --- | --- |
| **Left colectomy** | Laparoscopic | ICD-9-PCS | 17.35, 17.36 |
|  |  | ICD-10-PCS | 0DTM4ZZ, 0DTG4ZZ, 0DTN4ZZ  0DBM4ZZ, 0DBG4ZZ, 0DBN4ZZ |
|  | Open | ICD-9-PCS | 45.75, 45.76 |
|  |  | ICD-10-PCS | 0DTM0ZZ, 0DTG0ZZ, 0DTN0ZZ  0DBM0ZZ, 0DBG0ZZ, 0DBN0ZZ |
| **Right colectomy** | Laparoscopic | ICD-9-PCS | 17.32, 17.33 |
|  |  | ICD-10-PCS | 0DTF4ZZ, 0DTH4ZZ, 0DTK4ZZ  0DBF4ZZ, 0DBH4ZZ, 0DBK4ZZ |
|  | Open | ICD-9-PCS | 45.72, 45.73 |
|  |  | ICD-10-PCS | 0DTF0ZZ, 0DTH0ZZ,0DTK0ZZ  0DBF0ZZ, 0DBH0ZZ,0DBK0ZZ |
