# Supplementary material for: Association of frailty with outcomes of resection for colonic volvulus: A national analysis
Source: PLoS One. 2022 Nov 8;17(11):e0276917. doi: 10.1371/journal.pone.0276917 (PMC9642887; doi:10.1371/journal.pone.0276917)
Supplement: S2 Table — (DOCX) [file pone.0276917.s002.docx]

**S2 Table. Unadjusted outcomes for patients with sigmoid and cecal volvulus undergoing resection stratified by frailty**

| **Parameter** | **Sigmoid Volvulus** | | *P* | **Cecal Volvulus** | | *P* |
| --- | --- | --- | --- | --- | --- | --- |
|  | *nFrail*  (n=18,432) | *Frail*  (n=8,025) |  | *nFrail*  (n=33,920) | *Frail*  (n=6,390) |  |
| **Clinical Outcomes** |  |  |  |  |  |  |
| In-hospital mortality (%) | 5.7 | 10.6 | <0.001 | 3.6 | 10.3 | <0.001 |
| Perioperative hemorrhage (%) | 1.8 | 1.6 | 0.5 | 1.8 | 3.0 | <0.001 |
| Postoperative infections (%) | 3.0 | 3.8 | 0.04 | 3.1 | 5.1 | <0.001 |
| VTE (%) | 2.6 | 4.6 | <0.001 | 1.5 | 4.3 | <0.001 |
| Colostomy (%) | 36.9 | 57.2 | <0.001 | 1.6 | 3.2 | <0.001 |
| Ileostomy (%) | 2.4 | 2.2 | 0.7 | 5.0 | 14.3 | <0.001 |
| **Resource Utilization** |  |  |  |  |  |  |
| Length of stay (days, IQR) | 9 [7-14] | 13 [9-21] | <0.001 | 6 [5-9] | 12 [7-19] | <0.001 |
| Index hospitalization costs ($1,000s, IQR) | 24.8  [17.6-38.0] | 32.8  [22.3-54.2] | <0.001 | 17.9  [13.3-26.9] | 31.6  [20.0-54.3] | <0.001 |
| Non-home discharge (%) | 35.6 | 64.9 | <0.001 | 15.9 | 50.1 | <0.001 |
| 30-day non-elective readmission (%) | 13.9 | 16.6 | <0.001 | 11.2 | 16.9 | <0.001 |
